# Comparative analysis of package inserts of local and imported antihypertensive medications in Palestine

**DOI:** 10.1186/s12889-017-4782-x

**Published:** 2017-09-25

**Authors:** Sandra A. Qatmosh, Amer A. Koni, Baraa G. Qeeno, Dina A. Arandy, Maysa W. Abu-Hashia, Bahaa M. Al-hroub, Sa’ed H. Zyoud

**Affiliations:** 1 PharmD Program, College of Medicine and Health Sciences, An-Najah National University, Nablus, 44839 Palestine; 2 Poison Control and Drug Information Center (PCDIC), College of Medicine and Health Sciences, An-Najah National University, Nablus, 44839 Palestine; 3 Department of Clinical and Community Pharmacy, College of Medicine and Health Sciences, An-Najah National University, Nablus, 44839 Palestine

**Keywords:** Package inserts, Antihypertensive medications, Palestine

## Abstract

**Background:**

Package inserts (PIs) as a reliable reference for patients and health care providers should provide accurate, complete and up-to-date information. The purpose of the current study is to assess and compare the PIs of antihypertensive agents locally produced in Palestine and their imported counterparts.

**Methods:**

Thirty-five PIs were assessed for the presence of 31 information statements using a scoring method. Word counting of 20 headings and subheadings was used to evaluate and compare local and imported PIs for information quantity.

**Results:**

None of the analysed PIs fulfilled the criteria. All of them included the brand name, active ingredients, indications, directions for use, adverse drug reactions, drug–drug interactions, pregnancy and lactation considerations, and storage. Whereas none of them, either local or imported PIs, included the shelf life and instructions to convert tablets or capsules into liquid forms. Additionally, only one (5%) imported and no (0%) local PIs mentioned the duration of therapy. Moreover, 93.4% of local PIs were deficient in areas regarding the inactive ingredients and date of last revision, and 86.7% did not mention the drug dose and possibility of tablet splitting. Furthermore, the maximum dose was not indicated in 90% of imported and 86.7% of local PIs. In general, imported PIs contained more detailed information than their local counterparts, where the range of differences in medians between the local and imported PIs was from 1.5-fold for pregnancy considerations to >42.00-fold for the effect on the ability to drive and use machines.

**Conclusions:**

The findings of this study revealed the superiority of imported over local PIs in both quality and quantity of information provided. This emphasises the need for appropriate measures to be taken by the Ministry of Health and local manufacturers to ensure efficiency of local PIs in providing accurate, complete and up-to-date information.

## Background

The package insert (PI) is written information supplied with over-the-counter and prescribed medications to provide all necessary information for the patient or his/her caregivers about the drug [1, 2]. It is also considered one of the most useful tools to provide essential and scientific information to health care professionals [3]. In the 1960s, the Food and Drug Administration (FDA) required pharmaceutical companies to create leaflets that are oriented to the patients (patient PI) starting with isoproterenol inhalation. Then in 1970, oral contraceptives such as medroxyprogesterone acetate and diethylstilbestrol began being provided with PIs, due to the findings of an increased risk for thrombosis. Consequently, patients can make an informed decision to use or stop using these medications [4]. In the 1990s, the PI concept became widely accepted, and PIs were provided with many pharmaceutical preparations [5].

Educating patients about their prescribed medications is becoming an increasingly important aspect of health care [6]. The public has become increasingly conscious about their health and the medications received from pharmacists [7–9]. Physicians often provide patients with inadequate information regarding their medication usage and disease due to heavy patient load; and many patients may not be able to retain the verbal information given by their physicians for a long time.

Hypertension and medications used for the treatment of hypertension are increasingly prevalent in the Palestinian population [10–15], and information on the quality of PIs is imperative for ongoing efforts to improve hypertension management and its potential impact on public health. On the other hand, the number of poisoning incidents involving antihypertensive drugs reported to the American Association of Poison Control Centres’ toxic exposure surveillance system has progressively increased over the past 30 years [16, 17]. In addition, published data on poisoning by antihypertensive drugs in Palestine are sparse and our experience is gained mainly from Poison Control and Drug Information Centre indicated an increase in cases of antihypertensive exposures [18–20].

The information content of PIs should be clear, accurate, complete and updated continuously for the safe and effective use of medications and to avoid medication errors [2]. Globally, there is a little evidence on how PIs should be designed and written to meet the needs of intended users [21]. The quality and quantity of information in PIs are usually supervised by legislative health authorities.

In many countries, research has been conducted to evaluate the completeness of information provided within medication PIs. Some studies that evaluated PIs according to an evaluation criteria found that the analysed PIs did not provide uniform information regarding the safe and effective use of medications; and it was recommended to improve the content and the design of the existing PIs [3, 22]. Another study from India that evaluated the adherence of PIs to the guidelines of the regulatory authorities, showed that current PIs fail to adhere to the guideline criteria [23]. In Palestine, a study by Sawalha et al., [1] revealed significant differences in the quality and quantity of clinical information content provided between PIs of locally manufactured anti-infective medications and their imported equivalents.

Many patients are interested in increasing their knowledge about medications, but they are concerned about their use. Accordingly, most of them tend to read the PI to answer their questions and find the information they require [24]. Although several attempts have been done to improve information written in PIs, they were still criticised worldwide [1, 3, 25, 26]. A previous study in Palestine showed that more than 50% of patients read the product PIs, however, they found them unclear which further increased their concerns [27]. Indeed, it is important for pharmaceutical companies to follow an evidence-based guideline and international standards to create PIs with fully integrated content that suits the needs of these populations [21]. Also, these PIs have to be written and designed in a manner that is clear and easy to be comprehend and read [28]. Moreover, the limited sources to reach reliable, accurate and up-to-date information render the leaflet an important source of drug information [29].

This study, therefore, was carried out to evaluate and compare PIs of locally produced antihypertensive agents and their imported counterparts available in Palestine, where a combination of qualitative and quantitative approaches were used to analyse the data. The importance of this study is not only because it is the first in its kind in Palestine, but also due to the interest in its analysis since previous findings indicated that components of the leaflets were difficult for patients to understand [30, 31]. Additionally, this study offers some important insights into the quality of currently available PIs which can serve as a reference for the Palestinian Ministry of Health to impose strict rules and regulations on local companies to ensure provision of high-quality PIs.

## Methods

Hypertension and its cardiovascular complications are a major health issue due to their high prevalence [32], and one of the leading causes of mortality and morbidity in the Palestinian Territories [33]. For this reason, antihypertensive medications were selected to be the scope of the current study.

### Selection of PIs

Inclusion criteria for selecting the antihypertensive active ingredients were as follows: 1. manufactured by at least one local pharmaceutical company; 2. have at least one imported equivalent; 3. available in the West Bank/Palestinian local market; and 4. available in a solid oral dosage form (i.e. tablets and capsules).

Exclusion criteria included combination products and products that are unregistered in the pharmacy department of the Palestinian Ministry of Health. Based on those criteria, 11 active ingredients were selected and included: amlodipine, losartan, valsartan, spironolactone, furosemide, captopril, enalapril, atenolol, bisoprolol, carvedilol, and isosorbide-5-mononitrate, available as 35 leaflets; 15 of them locally manufactured and the remaining 20 are equivalent products manufactured abroad. The trade names of the local and imported antihypertensive medications included in the study are provided in Table 1.

**Table 1:** Trade names of the local and imported anti-hypertensive medications that were included in the study

Anti-hypertensive agent	Local products	Imported products
Carvedilol	Aricard®	Carvedilol-Teva® Carvedexxon®
Amlodipine	Vascopin® Amicor® Secure®	Norvasc® Amlodipine-Teva®
Losartan	Lozar®	Lotan® Losartan-Teva® Ocsaar® Losardex®
Bisoprolol	Hypocor®	Concor® Cardioloc®
Captopril	Cardiopril®	Aceril®
Enalapril	Anapril® Angiocare®	Enaldex®
Atenolol	Cortenol®	Normiten® Normalol®
Spironolactone	Spirone®	Aldacton® Spironolacton-Teva®
Isosorbed-5-mononitrate	Vasocor®	Monocord®
Furosemide	Urix® Diasix®	Fusid®
Valsartan	Valzan®	Diovan® Vector®

### Collection of PIs

The leaflets were collected mainly from pharmacies, where 20 pharmacies had conveniently (non-random) been selected to check for the availability of medications in the local market of the West Bank that were located primarily in Nablus, Hebron, Ramallah and Jenin.

### Evaluation of PIs

Collected PIs were evaluated and compared using a set of criteria derived from the literature [1–3, 27, 34, 35]. Additionally, all these criteria were selected based on FDA and European Medicines Agency requirement [36, 37]. A scoring method was used to assess the quality of information provided in each PI by checking for the availability of 31 information statements including: brand name; active ingredients; inactive ingredients (excipients); therapeutic class; mechanism of action; indications; drug dose; duration of use; missing dose; maximum dose; directions for use; overdose and management; warnings and precautions; effect on ability to drive and use machinery; contraindications; adverse drug reactions (ADR); drug–drug interactions; drug–food interactions; pregnancy considerations; lactation considerations; paediatric considerations; geriatric considerations; possibility of crushing and mixing with food or beverages; possibility of tablet splitting; instructions to convert tablets or capsules into liquid forms; pharmacokinetic information; shelf life; storage; name and address of manufacturers/distributors; date of last revision; and sources of information. For each one of the previously mentioned information statements, the PI receives a ‘1’ score if present and ‘0’ score if absent. The total score of each statement is then calculated for all antihypertensive agents from each one of the four local companies, for all local products together and for all imported products together. In addition, simple word counting is one of the most common methods for evaluation of PIs [1, 38–42]. Therefore, PIs were evaluated for the quantity of information provided by simple word counting of 20 headings and subheadings that were available in both local and imported PIs, whereas the remaining 11 criteria were excluded either due to unavailability in either local or imported PIs (*n* = 6), or where word counting of the criteria does not appear meaningful (*n* = 5). Three review authors (SQ, AK and BQ) independently selected the PIs, extracted the data and interpreted the data based on the eligibility criteria. Any disagreements between the review authors were discussed and resolved by consensus. No significant abstraction differences were seen between the reviewers.

### Statistical analysis

Results are expressed as frequencies and percentages, or as medians followed by interquartile ranges (IQR) in parenthesis. All statistical analyses were performed with the SPSS statistical package (SPSS 15.0; SPSS Inc., Chicago, Illinois). The normality of the data tested using Kolmogorov-Smirnov test. Since most of the variables did not follow a normal distribution, a non parametric test (i.e. Mann Whitney U test) was used to find out the statistical significance. To test differences in word counts of 20 headings and subheadings (e.g. inactive ingredients, therapeutic class, indications, drug dose, missing dose, maximum dose, directions for use, overdose and management, warning and precautions, effect on ability to drive and use machines, contraindications, adverse drug reactions, drug-drug interactions, drug-food interactions, pregnancy considerations, lactation considerations, pediatric considerations, possibility of tablet splitting, possibility of crushing and mixing with food or beverages, and storage) that were available in both local and imported PIs, the Mann Whitney U test was used with significance set at *p* < 0.05.

## Results

The PIs of local and imported products were analysed against 31 criteria, 11 out of 31 criteria were met in all local PIs compared to 14 out of 31 in the case of all imported PIs. None of the 35 leaflets achieved full adherence with these criteria, where it was found that the brand name, active ingredients, indications, directions for use, ADR, drug–drug interactions, pregnancy considerations, lactation considerations and storage were all mentioned in all local and imported PIs. However, instructions to convert tablets or capsules into liquid forms and shelf life were not mentioned in any of the PIs, either local or imported. Of all 35 PIs, only one (5%) imported PI provided information about the duration of use compared to none (0%) of the local PIs, which corresponds to only 2.8% of all PIs. Similarly, only two (10%) imported PIs explained in detail the pharmacokinetic information versus none (0%) of the local PIs, which represents only 5.7% of all PIs. However, 91–97% of the products have well covered the parts regarding overdose and management, warnings and precautions, contraindications, paediatric considerations, and name and address of manufacturers/distributors (Table 1).

On average, the PIs of imported products scored better than the local PIs regarding many topics, as shown in Table 2. In addition, it was noted that 93.4% of local PIs were deficient in areas regarding the inactive ingredients and date of last revision, 86.7% did not mention the drug dose and possibility of tablets splitting and 80% did not include the effect on the ability to drive and use machinery. In contrast, all PIs of imported products (100%) completely covered the inactive ingredients and the effect on the ability to drive, more than 70% included the date of last revision and the possibility of tablet splitting and 35% mentioned the drug dose. In addition, none of the 15 local PIs had any information about geriatric considerations and mechanism of action, while 55% and 30% of imported PIs provided detailed information about these topics, respectively. However, 90% of imported PIs and 86.7% of local PIs did not provide any information regarding the maximum dose. Interestingly, sources of information were found in 53% of the local products, while the PIs of the imported products did not mention any source of information. The information content of the PIs of the four local companies and imported products can be seen in Table 2.

**Table 2:** Content of information PIs provided with local and imported anti-hypertensive medications

Criteria	A N = 6 (40% of local PIs)	B *N* = 4 (26.67% of local PIs)	C *N* = 3 (20% of local PIs)	D *N* = 2 (13.33% of local PIs)	Total scores of Local Products *N* = 15 (42.86% of total PIs)	Total scores of Imported Products *N* = 20 (57.14% of total PIs)	Total *N* = 35 (100%)
Brand name	6 (100%)	4 (100)	3 (100)	2 (100)	15 (100)	20 (100)	35 (100)
Active ingredient	6 (100)	4 (100)	3 (100)	2 (100)	15 (100)	20 (100)	35 (100)
Inactive ingredients (excipients)	0 (0)	0 (0)	0 (0)	1 (50)	1 (6.6)	20 (100)	21 (60)
Therapeutic class	2 (33.3)	0 (0)	2 (66.6)	2 (100)	6 (40)	19 (95)	25 (71.4)
Mechanism of action	0 (0)	0 (0)	0 (0)	0 (0)	0 (0)	6 (30)	6 (17.1)
Indications	6 (100)	4 (100)	3 (100)	2 (100)	15 (100)	20 (100)	35 (100)
Drug dose	0 (0)	1 (25)	1 (33.3)	0 (0)	2 (13.3)	7 (35)	9 (25.7)
Duration of using	0 (0)	0 (0)	0 (0)	0 (0)	0 (0)	1 (5)	1 (2.8)
Missing dose	6 (100)	2 (50)	2 (66.6)	2 (100)	12 (80)	17 (85)	29 (82.8)
Maximum dose	0 (0)	1 (25)	1 (33.3)	0 (0)	2 (13.3)	2 (10)	4 (11.4)
Directions for use	6 (100)	4 (100)	3 (100)	2 (100)	15 (100)	20 (100)	35 (100)
Overdose and management	6 (100)	2 (50)	3 (100)	2 (100)	13 (86.6)	20 (100)	33 (94.2)
Warning and precautions	6 (100)	4 (100)	3 (100)	2 (100)	15 (100)	19 (95)	34 (97.1)
Effect on ability to drive and use machines	0 (0)	1 (25)	1 (33.3)	1 (50)	3 (20)	20 (100)	23 (65.7)
Contraindications	6 (100)	2 (50)	3 (100)	2 (100)	13 (86.6)	20 (100)	33 (94.2)
Adverse drug reactions	6 (100)	4 (100)	3 (100)	2 (100)	15 (100)	20 (100)	35 (100)
Drug-drug interactions	6 (100)	4 (100)	3 (100)	2 (100)	15 (100)	20 (100)	35 (100)
Drug-food interactions	1 (16.6)	1 (25)	1 (33.3)	1 (50)	4 (26.6)	19 (95)	23 (65.7)
Pregnancy considerations	6 (100)	4 (100)	3 (100)	2 (100)	15 (100)	20 (100)	35 (100)
Lactation considerations	6 (100)	4 (100)	3 (100)	2 (100)	15 (100)	20 (100)	35 (100)
Pediatric considerations	4 (66.6)	3 (75)	3 (100)	2 (100)	12 (80)	20 (100)	32 (91.4)
Geriatric considerations	0 (0)	0 (0)	0 (0)	0 (0)	0 (0)	11 (55)	11 (31.4)
Possibility of tablet splitting	0 (0)	1 (25)	0 (0)	1 (50)	2 (13.3)	15 (75)	17 (48.5)
Possibility of crushing and mixing with food or beverages	4 (66.6)	3 (75)	1 (33.3)	1 (50)	9 (60)	15 (75)	24 (68.5)
Instructions to convert tablets or capsules into liquid forms	0 (0)	0 (0)	0 (0)	0 (0)	0 (0)	0 (0)	0 (0)
Pharmacokinetic information	0 (0)	0 (0)	0 (0)	0 (0)	0 (0)	2 (10)	2 (5.7)
Shelf life	0 (0)	0 (0)	0 (0)	0 (0)	0 (0)	0 (0)	0 (0)
Storage	6 (100)	4 (100)	3 (100)	2 (100)	15 (100)	20 (100)	35 (100)
Name and address of manufacturers/distributors	6 (100)	4 (100)	3 (100)	2 (100)	15 (100)	19 (95)	34 (97.1)
Date of last revision	0 (0)	0 (0)	0 (0)	1 (50)	1 (6.6)	14 (70)	15 (42.8)
Sources of information	5 (83.3)	2 (50)	1 (33.3)	0 (0)	8 (53.3)	0 (0)	8 (22.8)

A word counting comparison was conducted for each criterion within the selected 20 criteria between the PIs of local and imported products (Table 3). As illustrated in Table 3, significant differences in the word counting were found in 13 criteria out of 20 criteria as follows: inactive ingredients (*p* < 0.001); therapeutic class (*p* = 0.016); indications (*p* = 0.023); warnings and precautions (*p* = 0.010); effect on ability to drive and use machinery (*p* = 0.002); contraindications (*p* < 0.001); ADR (*p* < 0.001); drug–drug interactions (*p* = 0.001); drug–food interactions (*p* = 0.001); pregnancy considerations (*p* = 0.010); paediatric indications (*p* = 0.019); possibility of tablets splitting (*p* < 0.001); and storage (*p* < 0.001). Additionally, it was noted that PIs of imported products scored higher than their local counterparts for all of the 13 criteria with significant *p* values (<0.05), where the range of differences in medians between the local and imported PIs was from 1.5-fold for pregnancy considerations to >42.00-fold for the effect on the ability to drive and use machinery. As an example of this difference, the median [Q1-Q3] for ADR word counting was 66.00 [62.00–97.00] words in all locally manufactured inserts compared to a median [Q1-Q3] of 334.00 [235.50–448.50] words in all imported inserts, which is about a five-fold difference in the median. In addition, similar findings were found regarding drug–drug and drug–food interactions, where in the case of drug–drug interactions the median [Q1-Q3] was 57.00 [49.00–73.00] words for local PIs compared to 151.00 [109.00–304.00] for imported PIs, which corresponds to a 2.62-fold difference in the median. In the case of drug–food interactions the median [Q1-Q3] was 0.00 [0.00–13.00] words for local PIs compared to 23.00 [12.00–35.00] for imported PIs, which corresponds to a > 23-fold difference in the median with superiority being attributed to imported PIs.

**Table 3:** Comparison between PIs of the local and imported products by word counting of 20 criteria

Variable	Local	Imported	P value^a^	Fold difference in medians
	Median [Q1-Q3]	Median [Q1-Q3]		
Inactive ingredients (excipients)	0.00 [0.00–0.00]	33.50 [20.00–58.50]	**0.000**	>33.50
Therapeutic class	0.00 [0.00–4.00]	5 [3.5–9.00]	**0.016**	>5.00
Indications	14.00 [10.00–20.00]	25.00 [18.50–37.00]	**0.023**	1.78
Drug dose	0.00 [0.00–0.00]	0.00 [0.00–108.5]	0.401	–
Missing dose	30.00 [15.00–34.70]	30.00 [26.00–34.50]	0.797	1.00
Maximum dose	0.00 [0.00–0.00]	0.00 [0.00–0.00]	0.898	–
Directions for use	37.00 [20.00–43.00]	33.00 [24.00–71.00]	0.365	1.12
Overdose and management	45.70 [38.00–71.00]	62.50 [44.00–80.00]	0.365	1.37
Warning and precautions	49.00 [36.00–173.00]	161.50 [117.50–322.00]	**0.010**	2.29
Effect on ability to drive and use machines	0.00 [0.00–20.50]	42.00 [33.00–47.00]	**0.002**	>42.00
Contraindications	28.33 [9.00–60.50]	89.00 [66.00–126.5]	**0.000**	3.14
Adverse drug reactions	66.00 [62.00–97.00]	334.00 [235.50–448.50]	**0.000**	5.06
Drug-drug interactions	57.70 [49.00–73.00]	151.00 [109.00–304.00]	**0.001**	2.62
Drug-food interactions	0.00 [0.00–13.00]	23.00 [12.00–35.00]	**0.001**	>23.00
Pregnancy considerations	16.70 [12.00–25.00]	25.00 [20.00–71.00]	**0.010**	1.50
Lactation considerations	13.00 [8.00–15.70]	16.00 [9.00–21.50]	0.116	1.23
Pediatric considerations	13.00 [6.70–55.00]	62.00 [39.50–72.50]	**0.019**	4.77
Possibility of tablet splitting	0.00 [0.00–0.00]	7.00 [4.50–12.00]	**0.000**	>7.00
Possibility of crushing and mixing with food or beverages	5.00 [1.50–5.00]	5.00 [3.00–7.00]	0.270	1.00
Storage	22.00 [15.00–35.00]	55.50 [47.50–59.00]	**0.000**	2.52

^a^The *p*-values are bold where they are less than the significance level cut-off of 0.05

## Discussion

Safe and effective use of medications is important for the success of the therapeutic process, and providing information that is accurate and reliable is considered essential to achieve such safe and effective use of both prescription-only and over-the-counter (OTC) medications. The information contained in medication leaflets is considered important, not just to the prescribers but also to the pharmacists and patients, where the written information allows the patient to be more involved in the treatment plan, judicially using the drugs, which increases their health literacy since counselling with a physician or pharmacist is mostly incomplete, forgettable and misunderstood by many patients [21, 28]. Moreover, it helps them to be aware of undesirable adverse effects and how to deal with or avoid these effects [21]. Recently, physicians and pharmacists have become more aware of the importance of PIs as patients’ outcomes and overall health costs may be decreased if they receive more information about their drugs [43]. In addition, patients’ adherence and contentment with their treatment plan will be improved when they receive high-quality PIs [44]. Therefore, it is important to evaluate the medication leaflets and subsequently ensure their optimisation to meet their purpose.

In our study, significant differences were found in both the quality and quantity of information provided in the PIs between the local and imported products, where local PIs were found to lack important types of information and were less detailed than their imported counterparts. Interestingly, most of the PIs either local or imported did not mention the duration of antihypertensive therapy as being lifelong or long-term, where it is included in only one imported leaflet (5%) and none (0%) of the local ones. This is a point of interest since patients may discontinue their antihypertensive medication on their own without referring to a health care provider, thus putting them at risk of serious complications such as stroke, myocardial infarction and many others. An eye-catching difference between the local and imported PIs can be demonstrated in the inactive ingredients criterion since we found it in only 6.6% of local PIs compared to 100% of the imported ones. In fact, it is worth mentioning the inactive ingredients as they may include sodium salts which may need to be counted with the daily sodium consumption since hypertensive patients are mostly sodium restricted, in addition to concerns of allergy in some patients. Moreover, date of last revision was missing in most of the local PIs, thereby affecting trust in the information provided.

Regarding the quantity of information included in the PIs, significant differences were found between the word counts of local and imported PIs. An obvious difference can be noted in the ADR criterion, where the imported products included much more detailed information compared to local products. Although all local PIs had good information about ADR, few of them provided verbal descriptions about these reactions whereas most of the imported PIs described ADR as being very common, common, uncommon, rare, or very rare and some of them included probabilities in describing these effects. Additionally, most of the products state ‘consult your doctor if you feel any change of your general health’ and do not provide advice on how to avoid or manage these ADR. Despite the conflicting research on the appropriate way to present ADR information to patients, whether by using numerical or verbal descriptions or both [45, 46], it is suggested by psychological studies that breaking large blocks of information (such as a continuous long list of ADR) into smaller groups and blocks results in better understanding and use of information [47]. Another important difference was found in the interaction part either drug–drug or drug–food interactions, where the median [Q1-Q3] of words for local PIs versus imported PIs was 57.00 [49.00–73.00] VS 151.00 [109.00–304.00] in the case of drug–drug interactions and 0.00 [0.00–13.00] VS 23.00 [12.00–35.00] in the case of drug–food interactions. This is especially important since many patients have multiple co-morbidities, are taking multiple medications and using OTC medications, therefore raising the probability of interactions between the medications either prescribed or OTC, in addition to interactions with food. For example, there is an increased risk of hyperkalaemia in the case of spironolactone, angiotensin converting enzyme inhibitors (ACEIs) and angiotensin receptor blockers (ARBs) when taken with potassium-rich food, e.g. bananas. However, no significant difference in word count was found regarding topics of directions for use and missing dose, and they were found in all local and imported PIs (100%) in the case of direction for use, and in more than 80% of local and imported PIs in the case of missing dose. This is promising since sufficient information on these areas is very important for the appropriate use of these medications and for achieving the desired goals of treatment.

In Palestine, the Palestinian Ministry of Health request PIs with all medications written in both English and Arabic languages, but there are no strict rules and regulations for monitoring the quality, quantity and design of the information [1]. A survey such as that conducted by Al-Ramahi et al. [48] has shown that a high proportion of consumers read the PI before using the medication. Al-Ramahi et al. concluded that quality and quantity of information in the PI must be optimised and regulated by the implementation of the appropriate measures by the Palestinian authorities and manufacturers [48]. In Saudi Arabia, all medications are required to be supplied with their original package and leaflet, similar to that submitted to the regulatory authority before marketing [3]. In Europe, every medication is required to be provided with understandable and readable PIs and written according to European Union law [49]. In Australia, computer-generated leaflets are the major patient information leaflet voluntarily supplied with medications, these leaflets have to be comprehensible to the reader and comparable to the PI according to Australian legislation [50]. Likewise, the United States (US) use computer-generated leaflets and the FDA requires that these leaflets should be accurate, understandable and well-designed [25].

These results seem to be consistent with those of other researchers who found that PIs are lacking headings and information necessary to ensure safe and effective use of medications [22, 35, 48]. Similar findings regarding the inactive ingredients, date of last revision and ADR were found in a previous study conducted in Palestine that compared the PIs of locally and imported anti-infective agents [1]. Another study carried out in India found that less than 25% of PIs included the date of last revision, self life, the effect on the ability to drive or use machinery and sources of information, which is consistent with our results [51]. In a study conducted to compare US, United Kingdom (UK) and Australian PIs, clear deficiencies in information regarding interactions, precautions and contraindications were found in the US PIs, whereas the Australian PIs were the best, followed by UK PIs [25].

The current study also found that a few leaflets were detailed regarding drug dose and pharmacokinetic information, in contrast to Ramdas et al. who demonstrated that Indian and multinational PIs completely covered these headings [2]. This is a point of interest since the inclusion of such information helps the prescribers to accurately choose the appropriate dose for each indication and can serve as a guide for dosage modifications in case of renal or liver impairment, or other affecting conditions.

In Palestine, a study conducted by Sweileh et al. showed that more than 50% of patients read the product PI. However, they found it unclear, which further increased their concerns, whereas 3.8% of patients either do not know or do not receive a patient PI [27]. Since the dissemination of information pertaining to the risk of medication such as the adverse effects of drugs, drug interactions or drug toxicity possesses a potential impact on public health, special attention should be placed on patient communication to reduce such risks. Furthermore, the medications comprise a ‘serious and significant’ public health concern for which patient information is required to ensure effective and safe use [6, 52, 53]. These findings emphasise the need for improving PIs to fulfil the criteria and to be updated and easy to understand by patients. Also, the supply of PIs should be mandatory, in addition to pharmacist counselling to ensure safe and effective use of medications.

## Strengths and limitations

The leaflets containing information are important to both pharmacists and patients since the right usage of clear, accurate, complete and up-to-date information information will lead to more compliance and adherence to medications. Antihypertensive medications are among the most often dispensed medications in pharmacies, due to the increasing population diagnosed with hypertension [32, 33, 54]. Our study is the first one to analyse, compare and discuss the content of antihypertensive medication leaflets in the local market of Palestine and one of the leading studies in the middle-east.

The most important limitation lies in the fact that until now, there is no standard evaluation criteria for PIs and the criteria used in the current study have advantages and disadvantages despite being used in previous studies. An additional limitation is that word counting may not always represent the quantity of information provided; where a larger word count does not always mean a greater amount of information. In addition, this study involved only one therapeutic class; antihypertensive agents and the availability of PIs was limited and determined by their availability in the West Bank since East Jerusalem and the Gaza Strip are difficult to reach due to political issues. Lastly, an issue that was not addressed in this study is the readability and comprehensibility of PIs, which is important to ensure so that patients can understand the information contained in PIs.

## Conclusions and recommendations

In conclusion, this study was conducted to evaluate and compare PIs of locally produced and imported antihypertensive agents using criteria derived from the literature. The most obvious finding is that significant differences were found between the two groups with superiority being attributed to imported PIs. Based on these findings and since the main purpose of PIs is to serve as a reliable source of information for the patient and as an assistive tool for health care providers, it is recommended that further research should be undertaken to include all therapeutic classes, extending to the remaining parts of Palestine, and assessing the readability and comprehensibility, besides evaluating the content. Moreover, the Ministry of Health and local manufacturers should pay greater attention to improve PIs and thus ensure their efficiency in providing accurate, complete and up-to-date information.

## Abbreviations

ACEIs: angiotensin converting enzyme inhibitors
ADR: adverse drug reactions
ARBs: angiotensin receptor blockers
e.g.: exempli gratia (means for example)
FDA: Food and Drug Administration
i.e.: id est. (means that is)
IQR: interquartile range
OTC: over-the-counter
PIs: Package inserts
SD: standard deviation
VS: versus
